# Novel MicroRNA Involved in Host Response to Avian Pathogenic Escherichia coli Identified by Deep Sequencing and Integration Analysis

**DOI:** 10.1128/IAI.00688-16

**Published:** 2016-12-29

**Authors:** Xinzheng Jia, Qinghua Nie, Xiquan Zhang, Lisa K. Nolan, Susan J. Lamont

**Affiliations:** a Department of Animal Genetics, Breeding and Reproduction, College of Animal Science, South China Agricultural University, Guangzhou, Guangdong, China; b Department of Animal Science, Iowa State University, Ames, Iowa, USA; c Guangdong Provincial Key Lab of Agro-Animal Genomics and Molecular Breeding and Key Laboratory of Chicken Genetics, Breeding and Reproduction, Ministry of Agriculture, Guangzhou, Guangdong, China; d Department of Veterinary Microbiology and Preventive Medicine, College of Veterinary Medicine, Iowa State University, Ames, Iowa, USA; University of California, Davis

**Keywords:** APEC, miRNA, chicken, pathology, spleen

## Abstract

Avian pathogenic Escherichia coli (APEC) causes one of the most common bacterial diseases of poultry worldwide. Effective control methods are therefore desirable and will be facilitated by a better understanding of the host response to the pathogen. Currently, microRNAs (miRNAs) involved in host resistance to APEC are unknown. Here, we applied RNA sequencing to explore the changed miRNAs and deregulated genes in the spleen of three groups of broilers: nonchallenged (NC), APEC-challenged with mild pathology (CM), and APEC-challenged with severe pathology (CS). Twenty-seven differentially expressed miRNAs (fold change >1.5; *P* value <0.01) were identified, including 13 miRNAs between the NC and CM, 17 between the NC and CS, and 14 between the CM and CS groups. Through functional analysis of these miRNA targets, 12 immune-related biological processes were found to be significantly enriched. Based on combined analyses of differentially expressed miRNAs and mRNAs within each of the three groups, 43 miRNA-mRNA pairs displayed significantly negative correlations (*r* < −0.8). Notably, gga-miR-429 was greatly increased in the CS group compared to levels in both the CM and NC groups. *In vitro*, gga-miR-429 directly repressed luciferase reporter gene activity via binding to 3′ untranslated regions of TMEFF2, NTRK2, and SHISA2. Overexpression of gga-miR-429 in the HD11 macrophage cell line significantly inhibited TMEFF2 and SHISA2 expression, which are involved in the lipopolysaccharide-induced platelet-derived growth factor (PDGF) and Wnt signaling pathways. In summary, we provide the first report characterizing the miRNA changes during APEC infection, which may help to shed light on the roles of these recently identified genetic elements in the mechanisms of host resistance and susceptibility to APEC.

## INTRODUCTION

Avian pathogenic Escherichia coli (APEC) causes one of the most common bacterial diseases of poultry, resulting in acute and mostly systemic infections in birds of various ages (1). After APEC-induced colibacillosis emerges in poultry farms, infected animals may suffer sudden death, septicemia, or localized inflammation, such as pericarditis and perihepatitis, in multiple organs, which may reduce egg production and feed conversion (growth) and thus lead to significant economic losses in poultry production (2). Although prophylactic measures based on vaccination are advisable, no fully effective vaccine against heterologous APEC strains has been produced to date (3). APEC also tends to be highly resistant to multiple antimicrobials, which has the potential to complicate treatment of animal and human disease (4–7). Therefore, to reduce economic losses in poultry production and to protect animal and human health, it is critical to understand the host immune response and resistance mechanisms against APEC infection.

Previous research suggested that the chicken is an excellent model organism to elucidate the pathogenic mechanisms of APEC (8). Several studies have examined the mRNA profiles in selected immune organs or cells of chickens exposed to APEC by using cDNA microarray or transcriptome sequencing (RNA-Seq) analysis (9–12). Microarray analysis of the transcriptome of chicken monocyte-derived macrophages revealed 1,603 genes modulated by APEC (9). In chicken spleen, 1,101 and 1,723 differentially expressed genes (DEGs) were found between an APEC-infected group showing severe pathology and a noninfected group at 1 day and 5 days postinfection, respectively (10). Also in other immune-related tissues such as bone marrow, bursa, and thymus, large numbers of significantly differentially expressed genes were detected between susceptible and resistant birds (13–15). These genes promoted the inflammation process mainly through several pathways, such as the Toll-like receptor, Jak-STAT, NOD-like receptor, p53, and cytokine signaling pathways (9–14, 16). Although these investigations have yielded a more refined assessment of the mRNA differences between the APEC-challenged group and controls, little is currently known about miRNA roles in host response and resistance to APEC infection.

MicroRNAs (miRNAs), a class of 20- to 23-nucleotide (nt) noncoding small RNAs, function as fundamental posttranscriptional regulators of several biological processes, including development, differentiation, organogenesis, growth control, and apoptosis. Moreover, deregulation of miRNA expression may contribute to diseases, for example, a variety of cancers (17). Several studies have demonstrated that miRNA expression profiles may be useful biomarkers for diagnostics, prognosis, and prediction of response to treatment and are powerful tools for disease prevention and therapeutics (18–20).

APEC infection occurs through the respiratory tract, including the gas exchange region of the lungs and the interstitium of the air sacs, and then invades the bloodstream, resulting in septicemia and colonization of multiple internal organs such as lung, liver, and spleen (1). The spleen, as the body's major blood filter, plays a major role in detecting cell damage during APEC infection and in APEC pathological mechanisms. Also, because of poorly developed lymphatic vessels and nodes in birds, the spleen, as the largest lymphatic organ, plays a greater role in immune function in avian than in mammalian species, including combining the innate and adaptive immune systems through lymphocyte generation, maturation, and storage (21, 22). Therefore, the spleen is able to induce an immediate innate reaction after recognizing pathogens and initiate an antigen-specific adaptive immune response via filtering antigens from the blood. Because of the spleen's crucial role in the systemic immune response, it would be helpful to better understand its genetic regulation of APEC infection and pathology. We previously investigated spleen mRNA in APEC-infected birds using RNA-Seq (12). To identify the miRNA-regulated genes responsible for host resistance and susceptibility to APEC infection, the same samples were used to detect miRNA changes by using high-throughput deep sequencing on spleens from three groups: nonchallenged (NC), resistant (challenged, mild pathology; CM), and susceptible (challenged, severe pathology; CS) broilers. Subsequently, based on combined analysis of miRNA and potential target mRNA expression profiles in spleen, we further characterized the miRNA-mRNA regulatory networks involved in the host response to APEC infection with the goal to better understand the mechanisms of APEC resistance and susceptibility.

## RESULTS

### Characterization of the deep-sequencing data.

After low-quality reads were filtered and adaptor sequences were trimmed, a total of 10,640,422 clean reads from the three small RNA libraries was obtained, including 3,462,706, 3,586,689, and 3,591,027 reads for the NC, CM, and CS groups, respectively. Through BLAST searches with the chicken genome, more than 80% of the reads could be perfectly mapped. To make the analysis computationally easier, the draft data were assembled by groups and formed into 255,260 unique NC sequences, 248,366 unique CM sequences, and 176,408 unique CS sequences, which were used for subsequent analysis. Among the three NC, CM, and CS groups, the percentages of matched clean reads were as high as 80.97%, 82.47%, and 88.59%, respectively, while the percentages of matched unique sequences were 77.56%, 73.00%, and 69.36%, respectively.

**FIG 1:**
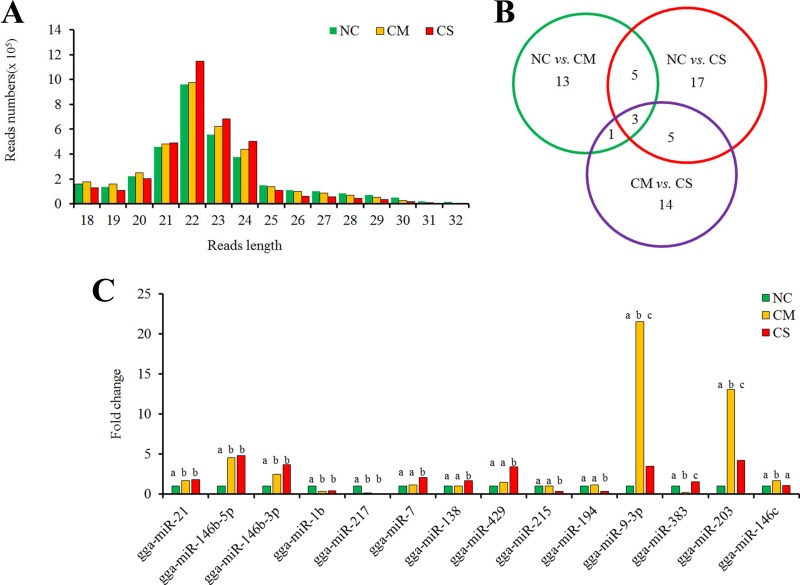
Different expression profiles of miRNAs among the NC, CM, and CS groups. (A) Size distribution of sequenced small-RNA reads. (B) Venn diagram demonstrates the overlap of differently expressed miRNAs among each group. Numbers in each section indicate the numbers of differently expressed miRNAs in the comparison. (C) The expression profiles of overlapped miRNAs with a significant change in at least two groups (*P* value <0.01, fold change >1.5). Different letters (a, b, and c) indicate statistically significant differences at a *P* value of <0.01. Only three miRNAs (gga-miR-383, gga-miR-203, and gga-miR-9-3p) were significantly differently expressed in each group. NC, nonchallenged control; CM, challenged, mild pathology; CS, challenged, severe pathology.

For all three groups, the distribution of the small RNA sequence length was mainly concentrated at 22 nt, followed by 23 and 21 nt (Fig. 1A), which is consistent with the known 21- to 23-nt range for miRNAs. For these small RNAs, about 75.94% unique sequences (9,790,738 sequences) were annotated based on the rfam database, and about 58% of them were defined as miRNAs. Other known categories of identified small RNAs (22%) include RNAs, rRNAs, tRNAs, snRNAs, and snoRNAs. These data suggested that, overall, miRNA fractions were effectively enriched from spleens in this study.

### Different expression profiles of miRNAs among groups differing in response to APEC.

A total of 153 known mature miRNAs were detected in at least one of the three groups (according to the miRbase, version 19.0) from chicken spleen. After DEG analysis, 27 miRNAs exhibited significantly different expression levels among three groups (fold change >1.5, *P* value <0.01, Benjamini *q* value <0.01), which included 13 miRNAs between the NC and CM, 17 between the NC and CS, and 14 between the CM and CS groups (Table 1; Fig. 1B). To validate the expression profiles, six miRNAs were confirmed by quantitative PCR (qPCR) (see Fig. 3). Except for the gga-miR-383 with a slight difference in the CM group, the expression patterns of gga-miR-21, gga-miR-146b-5p, gga-miR-146b-3p, gga-miR-429, and gga-miR-215 were comparable to the sequencing results. Thus, the expression profiles from the deep sequencing were reliable and appropriate for further analysis.

**TABLE 1:** Differential expression profile of splenic miRNAs among birds responding differently to APEC infection^*a*^

miRNA	No. of normalized reads per group		
	NC	CM	CS
gga-miR-21	429,813	722,377	785,763
gga-miR-146c	114,790	192,057	122,504
gga-miR-451	61,041	46,739	87,609
gga-miR-146a	10,131	17,679	13,472
gga-miR-146b-5p	2,504	11,378	12,076
gga-miR-144	2,998	2,391	3,924
gga-miR-215	2,108	2,162	727
gga-miR-147	1,093	1,524	1,754
gga-miR-7	266	304	553
gga-miR-33	357	275	428
gga-miR-146b-3p	126	311	460
gga-miR-499	249	173	340
gga-miR-138	109	111	181
gga-miR-194	126	144	42
gga-miR-383	102	20	157
gga-miR-34a	70	85	105
gga-miR-429	32	46	108
gga-miR-9-3p	7	150	24
gga-miR-1a	91	39	49
gga-miR-1b	95	33	42
gga-miR-301b	42	29	17
gga-miR-205a	0	52	15
gga-miR-203	4	46	15
gga-miR-122	11	26	15
gga-miR-135a	25	20	7
gga-miR-217	21	3	2
gga-miR-219	4	16	0

a Each library was normalized to obtain the expression level of transcripts per million reads using total clean read counts in this study. A fold change value of >1.5 of normalized reads and a *P* value of <0.01 among the NC, CM, and CS groups were considered to indicate differentially expressed miRNAs.

To investigate the host response and resistance to APEC infection in spleen, we have further analyzed the differences in expression levels between the resistant and susceptible groups. Eight miRNAs were significantly differently expressed in both the NC versus CM and the NC versus CS groups, and four miRNAs in both the NC versus CM and the CM versus CS groups, as well as eight in both the NC versus CS and the CM versus CS groups. Only three miRNAs (gga-miR-383, gga-miR-203, and gga-miR-9-3p) were significantly differently expressed in all three groups (Fig. 1B and C). To identify the major miRNAs responsible for host resistance, we specially focused on the differently expressed ones between the CM and CS groups. Although eight miRNAs displayed significant increases in both the NC versus CS and the CM versus CS groups, the expression levels of gga-miR-9-3p, gga-miR-383, and gga-miR-203 showed big changes in the CM but small changes in the CS group, while gga-miR-194, gga-miR-138, gga-miR-215 and gga-miR-7 exhibited significant changes in the CS versus CM group but very little difference in the CM versus NC group. Only gga-miR-429 had a consistently upregulated expression level, with about 1.4-fold in the CM versus NC group and 2.3-fold in the CS versus CM group (Fig. 1C). Thus, gga-miR-429 is a potential, novel candidate related to APEC resistance.

### Prediction of miRNA targets and their potential function in biological processes.

The ultimate function of miRNAs is dependent on the activity of target genes. Based on both TargetScan, version 6.2, and RNAhybrid systems, a total of 2,559 consensus potential miRNA targets were obtained for the 27 differentially expressed miRNAs among the NC, CM, and CS groups. Potential functional analysis of these genes showed that a total of 12 immune-related biological processes were significantly enriched (*P* value < 0.01), including leukocyte activation, lymphocyte activation, immune system development, leukocyte homeostasis, T cell activation, B cell activation, regulation of cytokine production, regulation of cytokine biosynthetic process, regulation of interleukin-6 production, immune effector process, leukocyte differentiation, and lymphocyte homeostasis (Table 2). The results suggest that the changed miRNAs may regulate these immune-related targets in the chicken spleen during APEC infection.

**TABLE 2:** Immune-related biological processes identified by gene ontology analysis of target genes^*a*^

Term	Description	No. of genes (% of total)	*P* value
GO:0002520	Immune system development	42 (1.37)	1.12E−04
GO:0045321	Leukocyte activation	32 (1.05)	1.13E−04
GO:0046649	Lymphocyte activation	29 (0.95)	1.35E−04
GO:0048534	Hemopoietic or lymphoid organ development	40 (1.31)	1.72E−04
GO:0042113	B-cell activation	15 (0.49)	4.30E−04
GO:0001817	Regulation of cytokine production	22 (0.72)	9.89E−04
GO:0001776	Leukocyte homeostasis	10 (0.33)	3.22E−03
GO:0042035	Regulation of cytokine biosynthetic process	14 (0.46)	3.91E−03
GO:0002521	Leukocyte differentiation	22 (0.72)	5.35E−03
GO:0042110	T-cell activation	19 (0.62)	5.47E−03
GO:0002252	Immune effector process	13 (0.42)	5.86E−03
GO:0030098	Lymphocyte differentiation	18 (0.59)	7.86E−03
GO:0002757	Immune response-activating signal transduction	9 (0.29)	8.37E−03
GO:0002764	Immune response-regulating signal transduction	9 (0.29)	8.37E−03

a The potential targets of 27 differently expressed miRNAs among the NC, CM, and CS groups significantly enriched biological processes (*P* < 0.01).

Different biological processes for host immune responses to APEC infection between the CM and CS groups were further characterized using KEGG pathway analysis, based on target genes of significant differentially expressed miRNAs among each group. In the NC versus CM group, four pathways were enriched (*P* value < 0.05), including regulation of actin cytoskeleton, cell cycle, selenoamino acid metabolism, and cysteine and methionine metabolism pathway; five pathways were enriched in the NC versus CS group, including progesterone-mediated oocyte maturation, purine metabolism, arginine and proline metabolism, oocyte meiosis, and lysosome pathway; seven pathways were enriched in the CM versus CS group, including regulation of actin cytoskeleton, lysosome, arginine and proline metabolism, alanine, aspartate and glutamate metabolism, oocyte meiosis, cell cycle, and focal adhesion. Among these pathways, the lysosome pathway, which promotes the capacity of macrophages to eliminate intracellular pathogens (23), was significantly activated only in CS samples (common in both the NC versus CS and the CM versus CS groups) (Table 3). These results indicated that different signaling pathways, including many non-immunity-related ones, were activated for resistant and susceptible responses to APEC infection.

**TABLE 3:** Enriched pathways by KEGG analysis among the NC, CM, and CS groups^*a*^

Class	Term	No. of genes (% of total)	*P* value	Fold enrichment
NC vs CS	Progesterone-mediated oocyte maturation	22 (0.8)	0.023	1.6
	Purine metabolism	34 (1.3)	0.027	1.4
	Arginine and proline metabolism	13 (0.5)	0.040	1.8
	Oocyte meiosis	26 (1.0)	0.041	1.5
	Lysosome	26 (1.0)	0.046	1.4
NC vs CM	Regulation of actin cytoskeleton	35 (2.0)	0.012	1.5
	Cell cycle	24 (1.4)	0.015	1.6
	Selenoamino acid metabolism	7 (0.4)	0.045	2.5
	Cysteine and methionine metabolism	9 (0.5)	0.048	2.1
CM vs CS	Regulation of actin cytoskeleton	44 (1.9)	0.007	1.4
	Lysosome	27 (1.2)	0.009	1.6
	Arginine and proline metabolism	13 (0.6)	0.022	2.0
	Alanine, aspartate and glutamate metabolism	10 (0.4)	0.025	2.2
	Oocyte meiosis	25 (1.1)	0.028	1.5
	Cell cycle	28 (1.2)	0.031	1.5
	Focal adhesion	41 (1.8)	0.039	1.3

a KEGG pathway analysis was performed with DAVID (https://david.ncifcrf.gov), which is based upon a Fisher Exact statistic methodology. The results were filtered using a *P* value of <0.05.

### miRNA-mRNA regulatory relationships in spleen after APEC infection.

**FIG 2:**
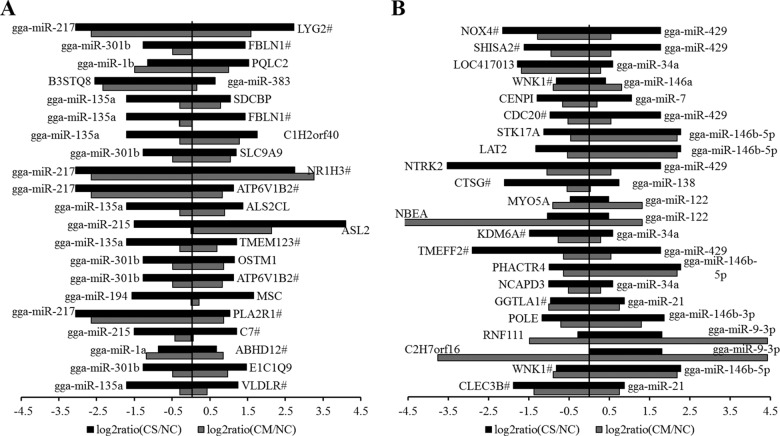
Integrated analysis of differently expressed miRNAs and mRNAs during APEC infection. (A) Negatively correlated expression of downregulated miRNAs and predicted targets. (B) Negatively correlated expression of upregulated miRNAs and predicted targets. The horizontal axis indicates the miRNA and mRNA ratio value of the CM versus NC and the CS versus NC groups, and the vertical axis indicates inverse expression pairs between miRNAs and mRNAs. The expression data were from the same samples of splenic tissues (nonchallenged control, challenged/mild pathology, and challenged/severe pathology groups) determined by Solexa sequencing. RNAhybrid system and TargetScan, version 6.2, were used to predict the miRNA targets. The Pearson correlation coefficient was used to estimate the expression relationships of miRNAs and mRNAs. Only miRNA-mRNA pairs with an *r* of less than −0.8 were considered to be strongly inversely correlated.

Most descriptions of miRNA function have focused on their roles as posttranscriptional regulators for target mRNAs. Because the impact of the miRNA is mostly due to the dampening of the steady state of the target mRNA, the expression levels of the direct target genes often show patterns that are the inverse of those of the corresponding miRNAs. Therefore, integrated miRNA and mRNA expression approaches were performed by pairwise correlation coefficient analysis to construct the miRNA-mRNA regulatory network. Finally, a total of 249 miRNA-mRNA pairs were identified, including 120 negative pairs and 129 positive ones (estimated by the Pearson correlation coefficient) (see Table S1 in the supplemental material). With the threshold of Pearson correlation coefficient values at −0.80, 43 negative pairs were chosen as the candidate miRNA and target pairs. Among them, 22 miRNA-mRNA pairs were associated with 10 upregulated miRNAs and 22 genes (Fig. 2A), including the following: gga-miR-21 regulating both CLEC3B and GGTLA1, gga-miR-146a/b regulating WNK1, gga-miR-138 regulating cathepsin G (CTSG), gga-miR-34a regulating KDM6A, and gga-miR-429 regulating TMEFF2, CDC20, NTRK2, SHISA2, and NOX4. On the other hand, in response to APEC infection, 21 miRNA-mRNA pairs were associated with 8 downregulated miRNAs and 19 genes (Fig. 2B), including the following: gga-miR-135a inducing very-low-density lipoprotein receptor (VLDLR), TMEM123, and FBLN1; gga-miR-1a inducing ABHD12; gga-miR-215 inducing C7; gga-miR-217 inducing PLA2R1, ATP6V1B2, NR1H3, and LYG2; and gga-miR-301b inducing ATP6V1B2 and FBLN1.

**FIG 3:**
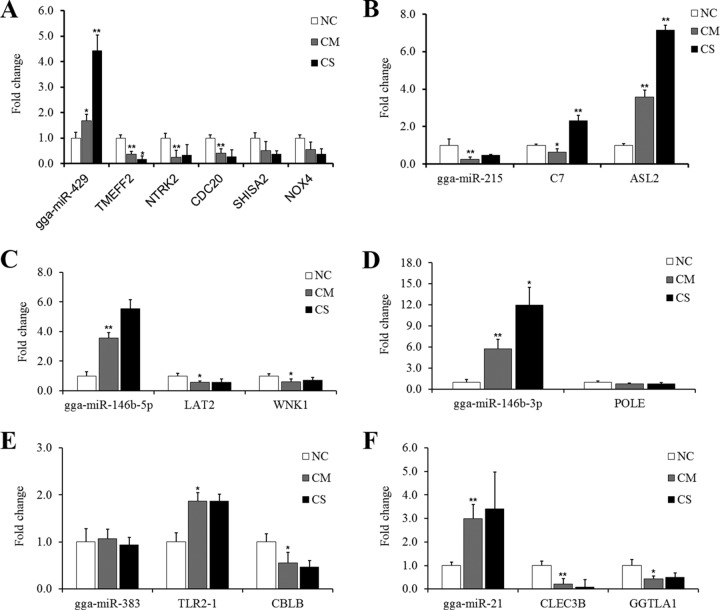
Quantitative reverse transcription-PCR detection of differentially expressed miRNA-mRNA pairs among the NC, CM, and CS groups. (A) gga-miR-429 versus target genes. (B) gga-miR-215 versus target genes. (C) gga-miR-146b-5p versus target genes. (D) gga-miR-146b-3p versus target genes. (E) gga-miR-383 versus target genes. (F) gga-miR-21 versus target genes. NC, nonchallenged control; CM, challenged, mild pathology; CS, challenged, severe pathology. U6 and β-actin genes were the host genes; the expression level of the NC group was normalized to 1.0. Fold change values were calculated using the comparative 2^−ΔΔ*CT*^ method [ΔΔ*C_T_* = Δ*C_T_*_(target gene)_ − Δ*C_T_*_(reference gene)_] from at least three independent experiments. The *P* values are indicated with asterisks when lower than 0.05 (*) or 0.01 (**) for the difference in results between the CM versus NC or the CS versus CM groups.

The negative expression correlations of miRNA-mRNA pairs were further confirmed by qPCR. The results showed that gga-miR-429 could influence five target genes, including TMEFF2, NTRK2, CDC20, SHISA2, and NOX4, in the spleen during APEC infection. Especially for TMEFF2 there was a significant, consistent decrease in expression in the CM (*P* value < 0.01) and CS (*P* value < 0.05) groups, which was the inverse of gga-miR-429 expression, where there was a consistent increase in the CM (*P* value < 0.05) and CS (*P* value < 0.01) groups (Fig. 3A). The change in gga-miR-215 expression was the inverse of that of its target gene ASL2 among the NC, CM, and CS groups (Fig. 3B). Similarly, gga-miR-146b-5p downregulated the expression of its target genes, including LAT2 and WNK1 (Fig. 3C). For gga-miR-146b-3p, the target gene, POLE, showed little change at the mRNA expression level (Fig. 3D). The gga-miR-21 expression level in the CM and CS groups was highly significantly different (*P* value < 0.01) and much higher than that in the NC group, respectively, while the expression of target genes (CLEC3B and GGTLA1) in the CM and CS groups was significantly (*P* value < 0.01 or *P* value < 0.05) lower than that in the NC group (Fig. 3F). Only for gga-miR-383 was there no significant difference (*P* value > 0.05) among the NC, CM, and CS groups (Fig. 3E).

### Validation of gga-miR-429 target genes *in vitro*.

**FIG 4:**
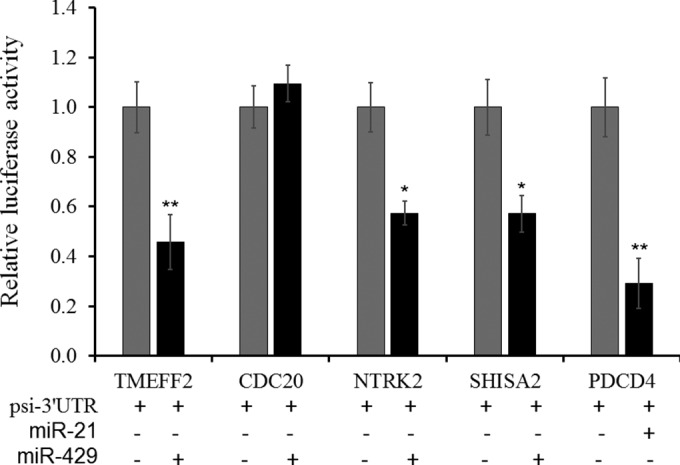
Validation of potential targets downregulated by gga-miR-429 in DF-1 cell lines. Cotransfections with miRNA mimic or negative oligonucleotides with psi-CHECK2-3′ UTR vectors of TMEFF2, SHISA2, NTRK2, and CDC20 genes in DF-1 cell lines were incubated for 36 h to detect Renilla luciferase activities. The vertical axis indicates the relative fold change of Renilla luciferase activities compared to the level of the negative group, using the firefly luciferase activity in the psi-CHECK2 vector as a normalization control. Each group was replicated as three wells. The *P* values were estimated by a *t* test and are marked with asterisks when lower than 0.05 (*) or 0.01 (**).

gga-miR-429 was significantly increased in the CS but not in the C group compared to the NC group. To further validate the potential targets of gga-miR-429 in the APEC infection process, we used dual-luciferase report systems to confirm the regulatory relationships. For the positive control, gga-miR-21 and the known targets of PDCD4 were detected in this study (24–26). After cotransfecting of DF-1 cells with gga-miR-429 (or a negative control) and psi-CHECK2 vectors containing 3′ untranslated regions (UTRs) of potential targets, the Renilla luciferase activities of TMEFF2, CDC20, and SHISA2 groups were greatly suppressed by 45%, 57%, and 57%, respectively. These results suggested that gga-miR-429 could mediate the transcription expression of TMEFF2, CDC20, and SHISA2 by directly targeting 3′ UTRs (Fig. 4).

### Overexpression of gga-miR-429 in chicken HD11 macrophage cells.

**FIG 5:**
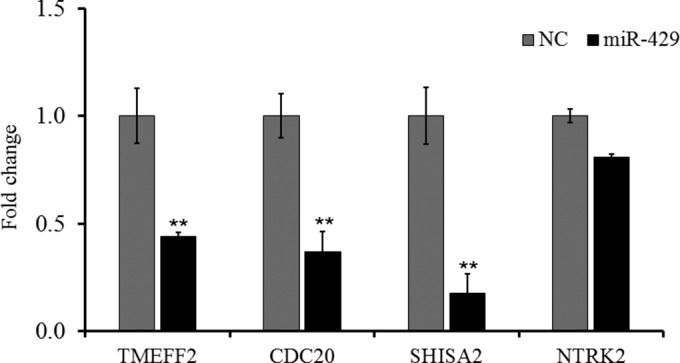
Overexpression of gga-miR-429 inhibits mRNA expression levels of TMEFF2 and SHISA2 in chicken HD11 macrophage cell lines. Mimics (100 μM) and control oligonucleotides were transfected into HD11 macrophage cells for 36 h to detect target gene expression levels. The fold change values were calculated using the comparative 2^−ΔΔ*CT*^ method [ΔΔ*C_T_* = Δ*C_T_*_(target gene)_ − Δ*C_T_*_(reference gene)_] from at least three independent experiments. The *P* values are indicated with asterisks when lower than 0.05 (*) or 0.01 (**) compared to results with the NC group.

To determine whether gga-miR-429 could downregulate the mRNA expression of these target genes in the immune system, a study of the HD11 macrophage-like cell line derived from bone marrow cells was performed *in vitro*. After 36 h of treatment with mimic miRNA, elevating gga-miR-429 significantly repressed the mRNA expression levels of TMEFF2, CDC20, and SHISA2 compared to the levels in the negative-control groups (*P* value < 0.05), while NTRK2 showed no significant change (Fig. 5). Combined with direct regulatory relationships identified by reporter gene systems, it is reasonable to suggest that gga-miR-429 directly inhibits TMEFF2 and SHISA2 transcription expression via binding to 3′ UTRs, whereas it indirectly inhibits CDC20 transcription expression via other mechanisms, which needs further investigation.

## DISCUSSION

MiRNAs have been proven to be strongly associated with pathogen invasion and host resistance (27). Therefore, it is necessary to identify and characterize the critical miRNAs in the chicken immune response to infectious diseases such as APEC, with the aim of understanding pathogenesis, improving animal welfare, reducing losses in poultry production, and keeping food safe. Here, Solexa sequencing, a high-throughout sequencing technology, was first used to detect the miRNA expression profile differences in chicken spleen after APEC challenge. In total, 153 known miRNAs were detected, including those expressed at low levels such as gga-miR-219 (normalized 4 and 16 reads for the NC and CM groups, respectively). Through DEG analysis, 27 miRNAs were found to be differentially expressed among the NC, CM, and CS groups, representing not only differences between infected and noninfected animals but also the degree of pathology resulting from infection. For these miRNAs, gga-miR-21 has the most abundant expression in both the CM and CS groups. Moreover, it was significantly upregulated in the CS and CM groups compared to the level in the NC group. Thus, we inferred that gga-miR-21 might have important effects on the lipopolysaccharide (LPS)-induced host immune response to APEC, which is consistent with the analysis of mouse gga-miR-21 (28, 29). Another highly expressed miRNA, gga-miR-146b-5p, was greatly increased in the CM and CS groups compared to the level in the NC group, but with no significant difference between the CS and CM groups, indicating that gga-miR-146b may have induced high expression after APEC infection. Except for these two miRNAs, other miRNAs with abundant expression showed no significant differences in two-way comparisons of the NC, CM, and CS groups. This suggests that these miRNAs might have no spleen-specific immune response to APEC and mainly reflect the general process of inflammation. In contrast, gga-miR-219 was detected only in the NC and CM groups, with a significant increase in the CM group but a decrease in CS group, which suggests that gga-miR-219 is related to pathology severity after APEC infection. This prediction is in agreement with the reports that high-dose challenges decreased miR-219-5p expression in human and mouse macrophages during the resolution of inflammation (30, 31). These results of our study also suggested that deep-sequencing technology benefits the discovery of functional miRNAs in APEC pathogenic processes, including miRNAs expressed at low levels. Also, in this study we defined three groups to detect miRNA expression according to the mild and severe pathology levels (or noninfected controls), which provides a useful approach to identify the candidate genes involved in host resistance to APEC. gga-miR-429 was detected as the most promising potential miRNA, which had a consistently upregulated expression level in the CM versus NC and CS versus CM groups.

It is useful to predict miRNA function and construct the regulation networks by the prediction of their targets and annotation of their biological functions. In this study, 12 immune-related biological processes were significantly enriched, including cytokine production and biosynthetic processes. Also, we found 23 genes enriched in the Toll-like receptor signaling pathway, which is consistent with a previous study by Sandford et al. (10) that the differentially expressed genes between APEC-infected and -noninfected chicken spleens on day 1 and on day 5 were significantly enriched in the Toll-like receptor and cytokine signaling pathways (10) (see Table S2 in the supplemental material). This highlights the potential function of the differently expressed miRNAs on the host response to APEC infection.

To identify the functional miRNAs related to host resistance, two sets of RNA-Seq data, the miRNA expression profile and previously reported transcriptome profiles (12), were integrated by this study. In total, 249 correlated miRNA and mRNA pairs were found, including 43 significantly negatively correlated pairs (*r* < −0.80). For the upregulated miRNAs, the most significantly correlated pair was gga-miR-21 and CLEC3B. Interestingly, gga-miR-21 was also an miRNA with the highest expression level in the spleen of all three (NC, CM, and CS) groups. CLEC3B has been recently reported as a candidate gene for osteoarthritis (32). Another target gene for miR-21, GGTLA1 (about 2-fold downregulated for the CM and CS groups), was reported to be involved in T-cell-mediated immune responses (33). gga-miR-135a and VLDLR were the most significantly correlated pair of the downregulated miRNAs. Small interfering RNA (siRNA) experiments revealed that VLDLR is critical for an efficient infection by rhinovirus 1B (34). The present study showed the same potential: gga-miRA-135a might enhance the function of VLDLR in resistance to APEC infection. gga-miR-138 was significantly downregulated in the CM group compared to the level in the NC group and may inhibit inflammation by enhancing CTSG, which has broad-spectrum antibiotic properties against Staphylococcus aureus, Klebsiella pneumonia and Escherichia coli (35–37). Interestingly, gga-miR-429 expression was considered to be highly related to APEC pathology, with 1.4-fold upregulation in the CM group and 3.4-fold upregulation in the CS compared to the NC group. Also, two direct target genes, TMEFF2 and SHISA2, were highly suppressed in spleen after APEC infection by 7.3-fold and 3.0-fold, respectively (*P* value < 0.01). Both genes are involved in the inflammatory response. TMEFF2 was reported to regulate both platelet-derived growth factor (PDGF) signaling and extracellular signal-regulated kinase (ERK) signaling pathways in several diseases. A recombinant form of the TMEFF2 ectodomain can suppress PDGF receptor signaling and promote ErbB4 and ERK1/2 phosphorylation via interaction with PDGF (38). The full-length form of TMEFF2 protein, acting as a receptor or coreceptor, promotes a strong ERK activation in response to growth factors (39). Moreover, the PDGF and ERK pathways played a great role in the LPS-induced inflammatory process. LPS exacerbated the lung fibrosis process by increasing PDGF-AA in macrophages and epithelial cells (40). LPS-elicited release of tumor necrosis factor alpha (TNF-α) was regulated in rat spleen via the ERK signaling pathway (41). ERK1-mediated posttranslational modifications controlled the process of LPS-mediated NLRP3 inflammasome priming (42). SHISA2, as another miR-429 target gene, was reported to be an important modulator of Wnt signaling in the chicken embryo, which enhanced host protection in response to enteric bacteria (43, 44). Therefore, it is reasonable to propose that miR-429 contributes to the APEC-induced pathology process and is involved in inflammasome-related PDGF, ERK, and Wnt signaling pathways through directly downregulating TMEFF2 and SHISA2 genes, respectively.

In conclusion, we present the first characterization of the splenic miRNA expression profile of the chicken in response to APEC infection. A total of 27 different expressed miRNAs were identified among three phenotypic groups representing noninfected chickens and infected chickens with severe pathology and those with mild pathology. Through integration analysis of differentially expressed miRNAs and mRNA, 43 miRNA-mRNA pairs displayed significantly negative correlations (*r* < −0.8). Specifically, we found that gga-miR-429, with a slow rise and sharp increase in the mild and severe pathology groups, could directly downregulate expression of TMEFF2 and SHISA2 mRNAs, both of which are involved in immune-related PDGF, ERK, and Wnt signaling pathways, by binding to the 3′ UTR. These investigations indicate that miRNAs play a major role in the APEC infection process. The findings will facilitate an understanding of resistance and susceptibility to APEC infection through miRNA-induced systems, provide guidance on potential vaccine targets, and may assist breeding for genetic resistance to APEC in poultry.

## MATERIALS AND METHODS

### Sample preparation.

Nonvaccinated, commercial male broilers were used. At 4 weeks of age, 240 birds were challenged by injecting the left thoracic air sac with 0.1 ml of APEC O1 (10^8^ CFU), and another 120 birds were injected with 0.1 ml of phosphate-buffered saline (PBS) as controls. Detailed information on the APEC O1 strain and challenge protocol were previously described (11). Necropsy was performed at 1 day postchallenge, and a summed lesion score ranging from 0 to 7 was determined for each APEC-challenged bird. Birds with lesions scoring 0 to 2 were classified as showing mild pathology, and those scoring 4 to 7 were classified as showing severe pathology. The mild and severe pathologies infer that birds were resistant and susceptible to APEC infection, respectively. Then, the total RNAs isolated from spleens of the three groups were subjected to Solexa deep sequencing to investigate the dynamics of chicken miRNA expression. The three pools of three spleens from each group were the same as those used in a previous study on mRNA expression (12). The APEC challenge experiment was performed at Iowa State University and approved by the Iowa State University Institutional Animal Care and Use Committee (approval 11-07-6460-G).

### Cell culture.

DF-1 chicken fibroblast cells were cultured in Dulbecco's modified Eagle medium (DMEM) with 10% fetal bovine serum (Gibco, Carlsbad, CA, USA). Chicken HD11 macrophage cells were cultured in RPMI 1640 medium containing 20 mM l-glutamine (Gibco, Carlsbad, CA, USA) and 10% fetal bovine serum (Gibco, Carlsbad, CA, USA). All cells were incubated at 37°C with 5% CO_2_.

### Total RNA isolation, small RNA library construction and Solexa sequencing.

For each group, three spleens were randomly chosen for analysis. Total RNA was isolated from each spleen using TRIzol (Invitrogen, Carlsbad, CA, USA) according to the manufacturer's instructions, and thereafter three pooled RNA samples (one per group) were prepared by quantification and pooling. Twenty micrograms of total RNA was purified by denaturing 15% PAGE for small-RNA enrichment (16 to 32 nt) and then ligated with sequencing adapters (Illumina, San Diego, CA, USA). Subsequently, cDNA was synthesized from small RNAs by reverse transcription and amplified with 15 PCR cycles to produce libraries. Amplified products were validated and quantified using an Agilent 2100 Bioanalyzer and a DNA 1000 Nano Chip Kit (Agilent Technologies, Santa Clara, CA, USA). Deep sequencing was performed on a Genome Analyzer IIx (Illumina, San Diego, CA, USA) by Shanghai Majorbio Bio-Pharm Biotechnology Co., Ltd. (Shanghai, China).

### Draft data analysis, assembly, and BLAST search of the chicken genome.

For all generated draft reads, low-quality bases (Sanger base quality of <20) of the 3′ end were trimmed using in-house perl scripts, and then the sequencing adapters were removed with the fastx toolkit software (http://hannonlab.cshl.edu/fastx_toolkit/). All identical sequences of sizes ranging from 16 to 32 nt were counted and eliminated from the initial data set. The assembled unique sequences were used for a BLAST search of the Rfam database, version 10.1 (http://rfam.sanger.ac.uk/), to remove non-miRNA sequences (rRNA, tRNA, snoRNA, etc.). Bowtie (http://bowtie-bio.sourceforge.net/index.shtml) was used to annotate the chromosomal location against the chicken genome data (Ensembl Trace Archive). Through a BLAST search of the miRbase, version 19.0 (http://www.mirbase.org/), the perfectly matched sequences were used to count and analyze the known miRNA expression profile.

### Analysis of differently expressed miRNAs.

Significantly different miRNA expression levels among the three groups were identified using the DEGseq package (45). First, each library was normalized to obtain the expression level of transcripts per million reads using total clean read counts. If the normalized miRNA reads were less than 20 in three libraries, that miRNA was removed from future differential expression analyses. The fold change value (>1.5) of normalized reads and *P* value (<0.01) were both considered to screen for differentially expressed miRNAs.

### Prediction of miRNA targets and GO and KEGG pathway analysis.

Regulation of gene expression by miRNAs is mostly through the interaction between mature miRNA and mRNA 3′UTRs. We downloaded the whole UTR database of chicken (http://www.ensembl.org/biomart/martview/) to predict the potential targets for differentially expressed miRNAs. RNAhybrid (http://bibiserv.techfak.uni-bielefeld.de/rnahybrid/) and TargetScan, version 6.2 (http://www.targetscan.org/), were used to predict the miRNA targets. Only the genes predicted by both methods and including more than a 7-bp seed match for miRNAs were considered reliable targets for further analysis. Gene Ontology (GO) and KEGG pathway enrichment were analyzed by DAVID, version 6.7 (http://david.ncifcrf.gov/), which is based upon a Fisher exact statistic methodology similar to that previously described (46). KEGG results were filtered using a *P* value of <0.05.

### Integration analysis between miRNA and mRNA data.

Two data sets of small-RNA sequencing and transcriptome sequencing were used for integrated analysis between miRNA and mRNA expression. Pearson correlation coefficients between the differentially expressed miRNAs and their target genes were computed using Excel software. The Pearson's *r* values were used to estimate the relationship (negative or positive correlation), and miRNA-mRNA pairs were considered to be strongly correlated with an *r* greater than 0.8 or less than −0.8.

### Validation of gga-miR-429 target genes through luciferase reporter systems.

The psi-CHECK2 vector (Promega, San Luis Obispo, USA) including double-luciferase reporter genes was used to test and validate the target sites for gga-miR-429, which was 3.4-fold upregulated in the CS group compared to the level in the NC group (no change between the CM and NC groups). The 3′ UTR fragments containing potential binding sites of four genes (TMEFF2, NTRK2, CDC20, and SHISA2) were cloned from chicken DNA samples. Then all the obtained sequences were inserted downstream of Renilla luciferase in the psi-CHECK2 vector (Promega, San Luis Obispo, USA) to generate the recombined dual-reporter vectors (Psi-3′UTR). To improve the accuracy of the experiment, the group of gga-miR-21 and PDCD4 were used as a positive control because it has been previously shown that gga-miR-21 negatively regulated PDCD4 through affecting binding in the 3′ UTR (47). Reporter experiments were performed in DF-1 cells using a mimic gga-miR-21 or gga-miR-429. After 0.35 μg of recombinant vector was cotransfected with 50 μmol of mimic miRNA per well of a 48-well plate into DF-1 cells for 36 h by Lipofectamine 2000 (Invitrogen, Carlsbad, CA, USA), luciferase activity was measured using a Dual-Luciferase Reporter Assay System (E1910) per the manufacturer's instructions (Promega, San Luis Obispo, USA). In each case, the constitutively expressed firefly luciferase activity in the psi-CHECK2 vector served as a normalization control for evaluating transfection efficiency.

### Overexpressed gga-miR-429 in chicken HD11 macrophage cells.

Mimic miRNA and a negative control synthesized by GenePharma (Shanghai, China) were used to overexpress gga-miR-429 in cell lines. Mimics (100 μM) and control oligonucleotides were transfected in HD11 macrophage cells using 12-well plates and Lipofectamine 2000 (Invitrogen, Carlsbad, CA, USA) per the manufacturer's instructions. After 36 h of transfection, the cells were harvested using TRIzol (Invitrogen, Carlsbad, CA, USA) to extract the total RNA.

### Quantitative real-time PCR.

After RNA samples were digested in solution with RNase-free DNase I (Life Technologies, Inc., Gaithersburg, CM, USA), they were quantified by a NanoDrop ND1000 instrument (NanoDrop Technologies, Wilmington, DE, USA). For each sample, 1 μg of total RNA was polyadenylated and reverse transcribed to cDNA using a miScript reverse transcription kit (Qiagen, Valencia, CA, USA), in which an oligo(dT) primer with a universal tag and miScript reverse transcription mix were included. A miScript SYBR green PCR kit (Qiagen, Valencia, CA, USA) and PCR master mix (SYBR green) kit (Toyobo, Japan) were used in quantitative PCR (qPCR) to determine the expression of mRNAs and miRNAs, respectively. The relative-quantification PCR was performed by a Bio-Rad CFX96 Real-Time PCR detection system (Bio-Rad Laboratories Inc., Hercules, CA, USA), and the relative mRNA level of each target gene was calculated using the comparative 2^−Δ*CT*^ method [Δ*C_T_* = *C_T_*_(target gene)_ − *C_T_*_(β-actin)_, where *C_T_* is threshold cycle]. Fold change values were calculated using the comparative 2^−ΔΔ*CT*^ method, in which ΔΔ*C_T_* = Δ*C_T_*_(target gene)_ − Δ*C_T_*_(reference gene)_. β-Actin and U6 genes were chosen as reference genes for mRNA and miRNA expression. All primers are described in Table S3 in the supplemental material. Three independent replications were used for each assay, and data are presented as means ± standard errors of the means (SEM). A Student *t* test was used to compare mRNA levels among different groups. The threshold for significance was set at a *P* value <0.05, and for high significance the threshold was set at a *P* value of <0.01.

### Accession number(s).

The three small RNA Solexa sequencing data sets are available in the Gene Expression Omnibus (GEO) database under accession number GSE51396.

## Supplementary Material

Supplemental material
