# Supplementary material for: Novel MicroRNA Involved in Host Response to Avian Pathogenic Escherichia coli Identified by Deep Sequencing and Integration Analysis
Source: Infect Immun. 2016 Dec 29;85(1):e00688-16. doi: 10.1128/IAI.00688-16 (PMC5203650; doi:10.1128/IAI.00688-16)
Supplement: Supplemental material [file supp_85_1_e00688-16__index.html]

Supplemental material 

# Novel MicroRNA Involved in Host Response to Avian Pathogenic Escherichia coli Identified by Deep Sequencing and Integration Analysis

## Supplemental material

- Supplemental file 1 -

  Table S1. Expression correlation of miRNA and target genes among NC, CM, and CS groups. Table S2. Twenty-three target genes enriched in Toll-like receptor signaling pathway. Table S3. Quantitative real-time RT-PCR primers for miRNA and target genes.

  PDF, 177K
