# Supplementary material for: Novel MicroRNA Involved in Host Response to Avian Pathogenic Escherichia coli Identified by Deep Sequencing and Integration Analysis
Source: Infect Immun. 2016 Dec 29;85(1):e00688-16. doi: 10.1128/IAI.00688-16 (PMC5203650; doi:10.1128/IAI.00688-16)
Supplement: Supplemental material [file IAI.00688-16_zii999091916s1.pdf]

Novel miRNA involved in host response to avian pathogenic *Escherichia coli* identified by deep sequencing and integration analysis

Xinzheng Jia[a,c], Qinghua Nie[a, c*], Xiquan Zhang[a, c], Lisa K. Nolan[d], Susan J. Lamont[b*]

[a]Department of Animal Genetics, Breeding and Reproduction, College of Animal Science, South China Agricultural University, Guangzhou 510642, Guangdong, China.

[b]Department of Animal Science, Iowa State University, Ames, Iowa 50011, USA.

[c]Guangdong Provincial Key Lab of Agro-Animal Genomics and Molecular Breeding and Key Laboratory of Chicken Genetics, Breeding and Reproduction, Ministry of Agriculture, Guangzhou 510642, Guangdong, China.

[d]Department of Veterinary Microbiology and Preventive Medicine, College of Veterinary Medicine, Iowa State University, Ames, Iowa 50011, USA.

[*]To whom correspondence should be addressed: Q. Nie (Tel. 86 20-85285759. Fax. +86 20-85280740. Email: nqinghua@scau.edu.cn) and S. J. Lamont (Tel. 1-515-294-4100. Fax. 1-515-294-2401. Email: sjlamont@iastate.edu).

Supplementary information.

All supporting data are included as additional files as follows: **Supplementary Table S1.** Expression correlation of miRNA and target genes among NC, MD and SV. miRNA-mRNA pairs of differently expressed miRNAs and mRNAs were evaluated for the regulatory relationships using Pearson correlation coefficient analysis. **Supplementary Table S2.** Twenty-three target genes regulated by differently expressed miRNAs were enriched in Toll-like receptor signaling pathway. **Supplementary Table S3.** Primers used in this study for quantitative real time RT-PCR and constructing recombinant vector.

Table S1. Expression correlation of miRNA and target genes among NC, CM and CS

miRNA	NC	CM	CS	Ensemble_id	Wikigenes	NC_FPKM	MD_FPKM	SV_FPKM	length	Pearson correlation coefficients
gga-miR-21	429812.8	722377.0	785763.2	ENSGALT00000019389	CLEC3B	287.7	112.6	80.0	2183	-0.9998
gga-miR-135a	24.5	19.6	7.3	ENSGALT00000016531	VLDLR	12.2	16.4	28.8	3230	-0.9995
gga-miR-203	3.5	45.8	14.7	ENSGALT00000015761		1.2	0.0	1.0	4486	-0.9994
gga-miR-301b-5p	42.0	29.4	17.1	ENSGALT00000031441		23.3	45.7	64.4	706	-0.9989
gga-miR-1a	91.1	39.3	48.9	ENSGALT00000013893	ABHD12	53.8	97.6	86.1	3144	-0.9969
gga-miR-146b	2503.8	11378.1	12075.5	ENSGALT00000021209		34.1	18.6	19.5	7106	-0.9932
gga-miR-215	2108.1	2162.4	726.6	ENSGALT00000023947	C7	13.4	9.9	30.9	3083	-0.9928
gga-miR-9*	7.0	150.5	24.5	ENSGALT00000019967	C2H7orf16	3.3	0.3	3.4	1038	-0.9902
gga-miR-9*	7.0	150.5	24.5	ENSGALT00000006632	RNF111	6.2	2.2	5.1	4300	-0.9881
gga-miR-217	21.0	3.3	2.5	ENSGALT00000033111	PLA2R1	24.3	44.4	49.5	5410	-0.9879
gga-miR-146b*	126.1	310.8	459.9	ENSGALT00000012422	POLE	7.7	4.7	3.4	7072	-0.9876
gga-miR-194	126.1	143.9	41.6	ENSGALT00000030884	MSC	3.3	3.3	10.5	1169	-0.9860
gga-miR-301b-5p	42.0	29.4	17.1	ENSGALT00000002628	ATP6V1B2	39.6	70.6	86.7	2885	-0.9846
gga-miR-301b	42.0	29.4	17.1	ENSGALT00000024702	OSTM1	25.3	46.0	56.3	2495	-0.9827

-5p										
gga-miR-21	429812.8	722377.0	785763.2	ENSGALT00000010504	GGTLA1	132.4	67.0	68.9	1930	-0.9813
gga-miR-34a	70.0	85.1	105.2	ENSGALT00000002261		17.3	12.2	8.8	5080	-0.9813
gga-miR-135a	24.5	19.6	7.3	ENSGALT00000036427	TMEM123	133.5	214.3	308.1	2531	-0.9798
gga-miR-146b	2503.8	11378.1	12075.5	ENSGALT00000004527		20.8	13.4	10.6	2902	-0.9787
gga-miR-215	2108.1	2162.4	726.6	ENSGALT00000039984	ASL2	0.2	1.0	4.1	1879	-0.9729
gga-miR-429	31.5	45.8	107.6	ENSGALT00000012579	TMEFF2	4.4	2.8	0.6	1225	-0.9705
gga-miR-34a	70.0	85.1	105.2	ENSGALT00000026141	KDM6A	11.7	6.9	4.3	4530	-0.9699
gga-miR-122	10.5	26.2	14.7	ENSGALT00000027557		1.3	0.0	0.7	8922	-0.9616
gga-miR-135a	24.5	19.6	7.3	ENSGALT00000039135		6.1	11.2	15.8	3293	-0.9605
gga-miR-217	21.0	3.3	2.5	ENSGALT00000002628	ATP6V1B2	39.6	70.6	86.7	2885	-0.9545
gga-miR-217	21.0	3.3	2.5	ENSGALT00000038464	NR1H3	0.0	9.5	6.7	2019	-0.9466
gga-miR-301b-5p	42.0	29.4	17.1	ENSGALT00000040527	SLC9A9	9.8	20.3	22.6	2890	-0.9399
gga-miR-135a	24.5	19.6	7.3	ENSGALT00000027122	C1H2orf40	19.4	46.9	65.5	773	-0.9373
gga-miR-122	10.5	26.2	14.7	ENSGALT00000039066	MYO5A	15.7	8.5	11.4	6934	-0.9317
gga-miR-135a	24.5	19.6	7.3	ENSGALT00000023014	FBLN1	2.0	1.6	5.5	3345	-0.9315
gga-miR-138	108.6	111.2	181.0	ENSGALT00000021823	CTSG	153.4	105.5	36.6	1061	-0.9256
gga-miR-429	31.5	45.8	107.6	ENSGALT00000020568	NTRK2	3.2	1.6	0.3	2962	-0.9146
gga-miR-205a	0.0	52.3	14.7	ENSGALT00000010112		68.0	31.9	44.4	2066	-0.9118
gga-miR-146b	2503.8	11378.1	12075.5	ENSGALT00000001987	LAT2	27.4	18.9	11.1	1540	-0.9069
gga-miR-135a	24.5	19.6	7.3	ENSGALT00000024899	SDCBP	146.8	251.9	302.0	1684	-0.9020

gga-miR-146b	2503.8	11378.1	12075.5	ENSGALT00000020177	STK17A	17.9	13.1	8.3	2841	-0.8966
gga-miR-144	2997.6	2391.4	3924.1	ENSGALT00000018368	WDFY3	7.2	12.4	6.0	10905	-0.8890
gga-miR-429	31.5	45.8	107.6	ENSGALT00000016208	CDC20	52.0	36.4	26.7	1650	-0.8862
gga-miR-383	101.6	19.6	156.6	ENSGALT00000016143		9.5	10.5	1.6	2939	-0.8651
gga-miR-7	266.1	304.2	552.9	ENSGALT00000008077	CENPI	9.4	6.0	3.9	3106	-0.8618
gga-miR-146a	10130.8	17678.9	13472.4	ENSGALT00000021209		34.1	18.6	19.5	7106	-0.8586
gga-miR-34a	70.0	85.1	105.2	ENSGALT00000013047	LOC417013	7.5	2.4	2.2	2356	-0.8346
gga-miR-429	31.5	45.8	107.6	ENSGALT00000027646	SHISA2	102.5	53.8	34.2	2668	-0.8326
gga-miR-1b	94.6	32.7	41.6	ENSGALT00000035550	PQLC2	14.4	28.8	41.6	756	-0.8125
gga-miR-301b-5p	42.0	29.4	17.1	ENSGALT00000023014	FBLN1	2.0	1.6	5.5	3345	-0.8124
gga-miR-429	31.5	45.8	107.6	ENSGALT00000027863	NOX4	3.3	1.4	0.8	2213	-0.8051

Tabel S2. 23 target genes enriched in Toll-like receptor signaling pathway

Gene ID	Description
ENSGALT00000015999	TANK-binding kinase 1
ENSGALT00000001405	chemokine (C-C motif) ligand 5; chemokine (C-C motif) ligand 4
ENSGALT00000039023	interferon regulatory factor 7
ENSGALT00000041052	interleukin 12B (natural killer cell stimulatory factor 2, cytotoxic lymphocyte maturation factor 2, p40)
ENSGALT00000019074	interleukin 8
ENSGALT00000019072	interleukin 8
ENSGALT00000015615	interleukin-1 receptor-associated kinase 4
ENSGALT00000025232	lymphocyte antigen 96

ENSGALT00000040001	mitogen-activated protein kinase 1
ENSGALT00000020182	mitogen-activated protein kinase kinase kinase 7 interacting protein 2
ENSGALT00000011897	mitogen-activated protein kinase kinase kinase 8
ENSGALT00000020111	nuclear factor of kappa light polypeptide gene enhancer in B-cells 1
ENSGALT00000016362	nuclear factor of kappa light polypeptide gene enhancer in B-cells inhibitor, alpha
ENSGALT00000008754	phosphoinositide-3-kinase, catalytic, beta polypeptide
ENSGALT00000039333	phosphoinositide-3-kinase, regulatory subunit 5, p101
ENSGALT00000011552	ras-related C3 botulinum toxin substrate 3 (rho family, small GTP binding protein Rac3); ras-related C3 botulinum toxin substrate 1 (rho family, small GTP binding protein Rac1)
ENSGALT00000037211	receptor (TNFRSF)-interacting serine-threonine kinase 1
ENSGALT00000012390	signal transducer and activator of transcription 1, 91kDa; signal transducer and activator of transcription 4
ENSGALT00000001612	toll-interleukin 1 receptor (TIR) domain containing adaptor protein
ENSGALT00000015034	toll-like receptor 2
ENSGALT00000011333	toll-like receptor 4
ENSGALT00000037374	toll-like receptor 6
ENSGALT00000026777	toll-like receptor 7
ENSGALT00000037443	v-akt murine thymoma viral oncogene homolog 1

Table S3. Quantitave real time RT-PCR primers for miRNA and target genes

Gene	forward primers	downward primers
TMEFF2	TTGAAGTCATGTCTTTGGGTCG	GCAGAAGCCATTGTAATGCTCT
NTRK2	ACAAGTAAGAATCCTGCCTCG	GCAAAGAATACCGTCAGTTCG
CDC20	GGCTTTGATGTAGAAGAGGCT	CCTACTGGAAGCAGGTGTAGTC
SHISA2	ACCAACTTCTCGGTGCTCAAC	GCCGGGTACATCTTCTGCTCT

NOX4	TTTCAGCAAAGGCACTATCCC	TTGTAGCACTTCCAGCCATCA
CTSG	AAACCGAAGTCTCCATCTACAGC	TCGTACCCGAAGGAAACAACA
TLR2-1	CAGAGCGAGTGGTGCAAGTAT	CTCCAGGTAGGTCTTGGTGTT
CBLB	TCTTACAGCATGGCAGGAATC	CCAACATGGGCATACTAAAGC
CLEC3B	AGAAGGTGAAGGTATTGGGTAA	CCCTAGAACTCCTAAAGTGGC
GGTLA1	AGTAAAGGCACAAGCCACATC	ATCCCAGTCTGGTTGGAATAA
C7	AGTGGCAAGGCGGAGACAGAT	TGAGACTGGGCTCGCACAAAA
ASL2	CCAATGAACGCAGACTGAAGG	TGCAGCAGGTGAGTGGAAATG
TLR4	GGAGGTTGTAGATTTGAGTGA	AAGGAGGAGAAAGACAGGGTA
LAT2	TGCGTCAGGTGCCAGCTCTAT	CCTCGCCAAGTGTTCGGGTTC
WNK1	GTGCGAGAAGAACAGGAGAAG	AACAGATGCTGAAGTAGTGGG
POLE	GGGAACTGATTGAGCAGATTG	CATAGCACCTGGGTAGGAGATT
PLA2R1	ACAGCAGCGGGCTCATTAGTA	CAATGTCCCTCAGGTCCTTGT
ATP6V1B2	CAACAGACAGATTTACCCACC	CTCACCAACTACAGCCTTCAT
NR1H3	GAGGACGATCAGGCACGGACAG	TTGCTTCGCGGTTATTAGGGT
LYG2	GTATTGAGCCTGCTGTGATTG	TATTCTCGTGCCCTGCCTGAT
U6	CGATACAGAGAAGATTAGCATGG	miScript Universal primer
gga-miR-21	TAGCTTATCAGACTGATGTTGA	miScript Universal primer
gga-miR-217	TACTGCATCAGGAACTGATTGGAT	miScript Universal primer
gga-miR-429	TAATACTGTCTGGTAATGCCGT	miScript Universal primer
gga-miR-135a	TATGGCTTTTTATTCCTATGTGA	miScript Universal primer
gga-miR-215	ATGACCTATGAATTGACAGAC	miScript Universal primer
gga-miR-146b	TGAGAACTGAATTCCATAGGCG	miScript Universal primer
gga-miR-146b*	CCCTATGGATTCAGTTCTGC	miScript Universal primer
NTRK2_UTR	CCGCTCGAGGGCTGTGTGAATGAATGTG	AGCTTTGTTTAAACTGGAGAGTCTGCTGATATAAC
TMEFF2._UTR	CCGCTCGAGATGCGGACTCAACTACATT	AGCTTTGTTTAAACCACTCAGGAACCACCATC

SHISA2_UTR	CCGCTCGAGCTGAGCGGAGGAGAAATACTT	AGCTTTGTTTAAACTTGTAATACGAGTTCACGGAGA
CDC20_UTR	CCGCTCGAGAGTTGGTGTTGTGGGAAG	AGCTTTGTTTAAACGCATAGGCTAAGAAGTTACTC
PDCD4_UTR	CCGCTCGAGTGGAACCAACTGCTGAAG	AGCTTTGTTTAAACGCTGGCTGAACATCTACA

Note: The reverse primers for miRNA are miScript Universal primer provided by the miScript SYBR Green PCR kit.
